# Diagnosis of functional strictures in patients with primary sclerosing cholangitis using hepatobiliary contrast-enhanced MRI: a proof-of-concept study

**DOI:** 10.1007/s00330-023-09915-3

**Published:** 2023-07-20

**Authors:** Sarah Poetter-Lang, Alina Messner, Nina Bastati, Kristina I. Ringe, Maxime Ronot, Sudhakar K. Venkatesh, Raphael Ambros, Antonia Kristic, Aida Korajac, Gregor Dovjak, Martin Zalaudek, Jacqueline. C. Hodge, Christoph Schramm, Emina Halilbasic, Michael Trauner, Ahmed Ba-Ssalamah

**Affiliations:** 1 Department of Biomedical Imaging and Image-Guided Therapy, Medical University Vienna, Vienna, Austria; 2 Department of Diagnostic and Interventional Radiology, Hannover Medical School, Hannover, Germany; 3 Department of Medical Imaging at the Beaujon University Hospital in Clichy, University of Paris, Clichy, France; 4 Division of Abdominal Imaging, Department of Radiology, Mayo Clinic, Rochester, MN USA; 5 Department of Gastroenterology, Hepatology, University Medical Center Hamburg – Eppendorf, Hamburg, Germany; 6 Division of Gastroenterology and Hepatology, Department of Internal Medicine III, Medical University of Vienna, General Hospital of Vienna (AKH), Vienna, Austria; 7 Department of Biomedical Imaging and Image-Guided Therapy, General Hospital of Vienna (AKH), Medical University Vienna, Waehringer Guertel 18-20, 1090 Vienna, Austria

**Keywords:** Cholangitis, sclerosing, Constriction, pathologic, Magnetic resonance imaging, functional, Contrast media, Cholangiopancreatography, endoscopic retrograde

## Abstract

**Objectives:**

PSC strictures are routinely diagnosed on T2-MRCP as dominant- (DS) or high-grade stricture (HGS). However, high inter-observer variability limits their utility. We introduce the “potential functional stricture” (PFS) on T1-weighted hepatobiliary-phase images of gadoxetic acid-enhanced MR cholangiography (T1-MRC) to assess inter-reader agreement on diagnosis, location, and prognostic value of PFS on T1-MRC vs. DS or HGS on T2-MRCP in PSC patients, using ERCP as the gold standard.

**Methods:**

Six blinded readers independently reviewed 129 MRIs to diagnose and locate stricture, if present. DS/HGS was determined on T2-MRCP. On T1-MRC, PFS was diagnosed if no GA excretion was seen in the CBD, hilum or distal RHD, or LHD. If excretion was normal, “no functional stricture” (NFS) was diagnosed. T1-MRC diagnoses (NFS = 87; PFS = 42) were correlated with ERCP, clinical scores, labs, splenic volume, and clinical events. Statistical analyses included Kaplan–Meier curves and Cox regression.

**Results:**

Interobserver agreement was almost perfect for NFS vs. PFS diagnosis, but fair to moderate for DS and HGS. Forty-four ERCPs in 129 patients (34.1%) were performed, 39 in PFS (92.9%), and, due to clinical suspicion, five in NFS (5.7%) patients. PFS and NFS diagnoses had 100% PPV and 100% NPV, respectively. Labs and clinical scores were significantly worse for PFS vs. NFS. PFS patients underwent more diagnostic and therapeutic ERCPs, experienced more clinical events, and reached significantly more endpoints (*p* < 0.001) than those with NFS. Multivariate analysis identified PFS as an independent risk factor for liver-related events.

**Conclusion:**

T1-MRC was superior to T2-MRCP for stricture diagnosis, stricture location, and prognostication.

**Clinical relevance statement:**

Because half of PSC patients will develop clinically-relevant strictures over the course of the disease, earlier more confident diagnosis and correct localization of functional stricture on gadoxetic acid-enhanced MRI may optimize management and improve prognostication.

**Key Points:**

• *There is no consensus regarding biliary stricture imaging features in PSC that have clinical relevance.*

• *Twenty-minute T1-weighted MRC images correctly classified PSC patients with potential (PFS) vs with no functional stricture (NFS).*

• *T1-MRC diagnoses may reduce the burden of diagnostic ERCPs.*

**Graphical abstract:**

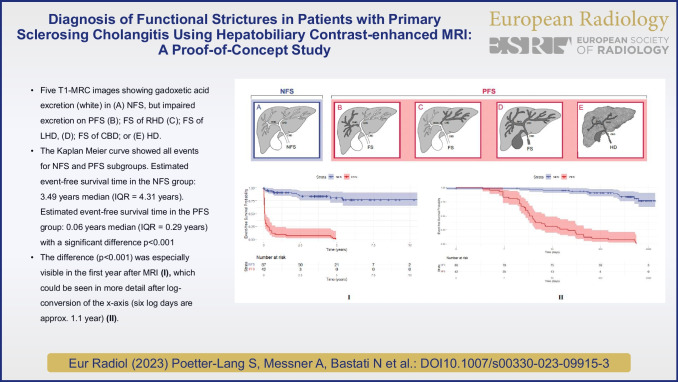

**Supplementary information:**

The online version contains supplementary material available at 10.1007/s00330-023-09915-3.

## Introduction

Primary sclerosing cholangitis (PSC) is a chronic, idiopathic, fibroinflammatory cholestatic liver disease that causes strictures of the intra- and/or extrahepatic biliary tree leading to progressive biliary and hepatic damage that results in portal hypertension and hepatic dysfunction (HD) within 10 to 15 years after the initial diagnosis [1]. Orthotopic liver transplantation (OLT) is the only effective treatment [1]. PSC patients are also prone to develop cholangiocarcinoma (CCA), gallbladder carcinoma, and, in the setting of cirrhosis, hepatocellular carcinoma (HCC). Furthermore, those with concurrent IBD are at risk of colorectal cancer [2]. Because half of PSC patients will develop a so-called dominant stricture (DS) over the course of the disease, it is imperative to diagnose such a stricture early, and to determine its location, as well as its clinical significance, to optimize management and render a prognosis [3, 4].

Both the American Association for the Study of Liver Diseases (AASLD) and the European Association for the Study of the Liver (EASL) guidelines define a DS as a lumen diameter ≤ 1.5 mm in the common bile duct (CBD) or ≤ 1 mm in either the main right (RHD) or left hepatic duct (LHD) within 2 cm of the hilum, as measured on endoscopic retrograde cholangiopancreatography (ERCP) [5, 6]. However, applying these cutoffs to conventional T2-weighted magnetic resonance cholangiopancreatography (T2-MRCP) [7] has been unsuccessful due to technical differences in image acquisition [8]. To address this problem, stricture severity, defined as either ≥ 75% (high-grade stricture, HGS) or < 75% (low-grade) narrowing of the CBD or hepatic duct lumen [8, 9], was introduced specifically for T2-MRCP. Even so, despite being defined for T2-MRCP, the morphologic appearance of HGS provides no more information about the clinical relevance of a stricture than DS [4, 7, 8]. However, it remains to be seen if the term HGS, which was accepted into the PSC imaging lexicon in 2021, will be widely adopted by radiologists and referring physicians [8].

Thus, recently, the consensus opinion of the International PSC Study Group defined a DS as possible if a segment of the extrahepatic or first-order intrahepatic ducts is narrowed on high-quality T2-MRCP or ERCP with either: (a) a 2-month history of worsening cholestatic symptoms plus recent, elevated bilirubin and/or alkaline phosphatase level(s) 1.2 × above baseline; or (b) elevated bilirubin and/or alkaline phosphatase level(s) 1.5 × above baseline within the previous 6 months [10]. However, this is a very controversial point. According to the recent MR Working Group of the International Primary Sclerosing Cholangitis Study Group, the term DS should not be used with MRCP. The study group recommends using the term HGS, which refers to the morphology of the duct(s) [8]. Regardless, the triad of clinical, laboratory, and radiologic features, although suggestive [7, 8], precludes confident, accurate diagnosis that would enable timely intervention to avert OLT and/or mortality.

To date, no universally accepted nomenclature defines a diagnostic biliary stricture of potential prognostic relevance. An ideal definition would be based on an objective, easily reproducible feature with high inter-reader agreement. Therefore, we propose the term “potential functional stricture” (PFS), defined as impaired gadoxetic acid (GA) excretion on 20-min, T1-weighted hepatobiliary contrast-enhanced MR cholangiography (T1-MRC). GA (Primovist® in Europe, Eovist® in the USA), normally taken up by functioning hepatocytes, then excreted via the bile ducts and kidneys within 20 min, allows visualization of physiologic bile excretion [11]. GA-MRI, including T1-MRC, has shown high sensitivity and specificity in diagnosing various biliary disorders, e.g., PSC and post-OLT anastomotic strictures [11–14].

We chose the term “potential functional stricture” (PFS) consciously, as impaired gadoxetic acid excretion can be due to either a true functional stricture (FS), i.e., significant bile duct narrowing with mechanical blockage or to hepatocellular dysfunction (HD) that is characteristic of advanced PSC. If “potential functional stricture” (PFS) is diagnosed, T2-MRCP, in addition to all-sequence MR, can help distinguish significant biliary obstruction, i.e., true FS, from HD of advanced PSC, as previously reported [15, 16]. A pruned tree appearance [17] and features of liver cirrhosis and/or portal hypertension are consistent with HD, once dilated bile ducts due to biliary obstruction can be excluded, as previously described by the functional liver imaging score [18–20]. In ambiguous cases or if clinically indicated, ERCP can be additionally performed.

Therefore, by determining if excretion is normal (i.e., no functional stricture (NFS)) or impaired (i.e., PFS) on the HBP of GA-MRI, using this binary system, we had two aims: to assess inter-reader agreement and prognostic value of PFS diagnosis and location on T1-MRC versus DS or HGS diagnosis and location on T2-MRCP in PSC patients, using ERCP as the gold standard.

## Materials and methods

### Patients

**Fig. 1:**
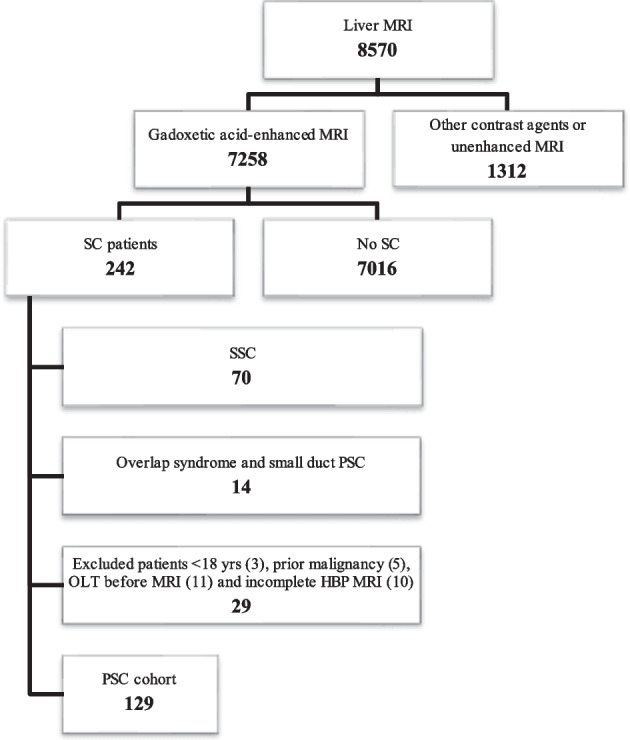
Flowchart. Between 2007 and 2022, 7258 patients underwent a standardized 3.0-Tesla gadoxetic acid-enhanced MRI of the liver. Of these, 242 had liver pathology other than sclerosing cholangitis (SC). Seventy patients were excluded for suspicion of secondary SC. Of the remaining 172 patients who met the criteria for primary sclerosing cholangitis (PSC), 14 had concurrent autoimmune hepatitis (i.e., overlap syndrome) and/or small duct PSC. We excluded another 29 PSC patients for the following: three were under 18 years of age, five had prior malignancy, 10 had an incomplete HBP MRI, and 11 had had previous OLT. Therefore, the final study cohort consisted of 129 patients

Our institutional review board approved this retrospective, single-center study. All patients gave written, informed consent for MRI and interventional procedures. Only patients with confirmed PSC according to EASL guidelines [5, 6] who underwent GA-MRI between Oct 2007 and March 2022 were included. We excluded patients with secondary sclerosing cholangitis, small-duct PSC, or confounding liver illnesses and who were under the age of 18 and/or had incomplete GA-MRI exams. Autoimmune hepatitis/PSC overlap syndrome, and other concomitant liver diseases, such as primary biliary cholangitis (PBC), hepatitis B or C infection, Wilson’s disease, haemochromatosis, autoimmune hepatitis, alcoholic liver disease, NAFLD/NASH, previous orthotopic liver transplantation OLT, previous choledochojejunostomy, cholangiocellular carcinoma, hepatocellular carcinoma, and cirrhotic decompensation, at the time of inclusion were considered confounding liver illnesses [6]. Importantly, the diagnosis of PSC was made only after potential causes of secondary sclerosing cholangitis (SSC) had been ruled out. These included IgG4-related cholangitis (IRC), sclerosing cholangitis of the critically ill patient (SC-CIP), or surgical/mechanical, toxic/drug-induced, infectious, and other immune-mediated and ischemic etiologies [21] (Fig. 1).

### Clinical data

Demographic and clinical data obtained from electronic medical records included patient age, gender, body mass index (BMI), date of and indication for MRI, follow-up imaging exams, duration of PSC (the time from initial PSC diagnosis to the end of the study in March 2022), and the presence of liver cirrhosis or inflammatory bowel disease. The Baveno consensus [22] was used to determine liver cirrhosis, including the presence of portal hypertension diagnosed by clinical, laboratories, elastography, imaging criteria, and occasionally histology post-biopsy. Routine surveillance in all PSC patients was conducted according to the EASL CPG and European Crohn’s and Colitis Organization (ECCO) guidelines, i.e., all cohorts underwent a full ileocolonoscopy with histology to exclude concurrent IBD with a recall within 2–4 years when the index colonoscopy was negative [6]. Current therapy and clinical findings within 6 months of GA-MRI were also recorded (Table 1). Laboratory tests performed within 2 weeks of MRI, plus clinical scores that indicated disease severity, including MELD, Revised Mayo-Risk-Score (RMRS), Fib-4, APRI, ALBI, UK-PSC risk scores, and Prognostic Index of the Amsterdam-Oxford model (PI-AOM), were recorded (Table 2).

**Table 1:** Patient characteristics, primary and secondary endpoints, and comorbidities of the two diagnostic groups (normal (*NFS*) vs. impaired excretion (*PFS*)) based upon the hepatobiliary phase T1-MRC of gadoxetic acid–enhanced MRI

Main characteristics Total number of patients *n* = 129	Normal excretion (no functional stricture) *n* = 87	Impaired excretion (potential functional stricture) *n* = 42	*p*-value
Mean age (years)	39.8 ± 14.3	44.6 ± 15.3	0.090
Female	38 (43.7%)	14 (33.3%)	0.262
Male	49 (56.3%)	28 (66.7%)	
Mean BMI (kg/m^2^)	23.7 ± 4.4	23.4 ± 4.4	0.695
Ulcerative colitis	32 (36.8%)	17 (40.5%)	0.685
Crohn’s disease	22 (25.3%)	11 (26.2%)	0.912
Ursodiol	87 (100%)	42 (100%)	**-**
Events			
Primary endpoints			
Death	4 (4.6%)	3 (7.1%)	0.682
Liver transplantation	5 (5.7%)	14 (33.3%)	** < 0.001**
Secondary endpoints			
ERCP-guided treatment	0 (0%)	36 (85.7%)	** < 0.001**
Variceal bleeding	3 (3.4%)	3 (7.1%)	0.390
Encephalopathy	0 (0%)	2 (4.8%)	0.104
CCA and gallbladder cancer	4 (4.6%)	2 (4.8%)	1.000
HCC	1 (1.1%)	1 (2.4%)	0.547
Comorbidities			
Fatty liver disease	0 (0%)	1 (2.4%)	0.326
Cholecystectomy	1 (1.1%)	2 (4.8%)	0.247
Arterial hypertension	2 (2.3%)	6 (14.3%)	**0.015**
Diabetes	1 (1.1%)	1 (2.4%)	0.547

*BMI* body mass index, *CCA* cholangiocarcinoma, *ERCP* endoscopic retrograde cholangiopancreatography, *HCC* hepatocellular carcinoma, *OLT* orthotopic liver transplant

Data presented for the means ± standard deviation or absolute numbers and percentage of the group

The values that are bolded are the ones that have *p*<0.05, i.e., are statistically significant

**Table 2:** Correlation between clinical scores, splenic volume, laboratory findings, and T1-MRC diagnoses

Score/parameter	T1-MRC diagnoses		*p*-value
	Normal excretion (no functional stricture)	Impaired excretion (potential functional stricture)	
MELD	3.09 ± 4.23	7.29 ± 6.39	**0.006**
Revised Mayo Risk Score	− 0.63 ± 1.01	0.82 ± 1.45	** < 0.001**
FIB4 index	0.86 (0.89)	1.17 (2.63)	0.031
APRI	0.32 (0.38)	0.57 (1.14)	**0.015**
ALBI	− 3.00 ± 0.48	− 2.27 ± 0.78	** < 0.001**
Short‐Term UK‐PSC Risk Score	− 3.36 ± 0.53	− 2.42 ± 1.12	** < 0.001**
Amsterdam-Oxford model for PSC	1.49 ± 0.80	2.01 ± 0.98	**0.001**
Spleen volume (cm^3^)	294 (190)	432 (416)	**0.016**
Thrombocytes (10^9^/L)	264 ± 120	242 ± 129	0.354
Hematocrit (%)	39.3 ± 4.27	36.3 ± 5.68	**0.003**
Hemoglobin (g/dL)	13.4 ± 1.65	12.2 ± 2.02	** < 0.001**
Albumin (g/mL)	43.2 ± 5.17	38.6 ± 6.41	** < 0.001**
Bilirubin (mg/dL)	0.63 (0.64)	1.78 (4.14)	** < 0.001**
Creatinine (mg/dL)	0.79 (0.21)	0.78 (0.20)	0.669
GGT (U/L)	114 (204)	214 (225)	**0.006**
GOT (U/L)	35.0 (34.0)	62.0 (58.5)	** < 0.001**
GPT (U/L)	39.0 (45.0)	64.5 (59.5)	**0.004**
AP (U/L)	146 ± 135	316 ± 253	** < 0.001**
Triglyceride (mg/dL)	88.0 (45.5)	108 (79.0)	**0.020**
Glucose (mg/dL)	93.9 ± 22.5	96.6 ± 15.8	0.509
CRP (mg/L)	0.38 (0.99)	0.63 (1.72)	0.046
Fibrinogen (mg/dL)	381 ± 101	428 ± 113	**0.025**
CA19-9 (U/mL)	12.3 (19.0)	26.1 (61.2)	**0.017**
CEA (ng/mL)	1.40 (2.08)	2.00 (1.10)	0.396
AFP (ng/mL)	1.85 (1.75)	1.85 (0.88)	0.730

Values are expressed as mean ± standard deviation or median (interquartile range)

^*^Student’s *t*-test (Welch corrected in case of unequal variances) or Wilcoxon rank-sum test

*MELD* model for end-stage liver disease, *FIB 4* Fibrosis-4 Index for Liver Fibrosis, *APRI* AST-to-platelet ratio index, *ALBI* albumin-bilirubin, *GGT* gamma-glutamyl transferase, *GOT* glutamic oxaloacetic transaminase, *GPT* glutamate pyruvate transaminase, *AP* alkaline phosphatase, *CRP* C-reactive protein, *CA 19–9* carbohydrate antigen 19–9, *CEA* carcinoembryonic antigen, *AFP* alpha-fetoprotein, *AST* serum aspartate aminotransferase level

MELD Score: The MELD score predicts the short-term and overall survival after liver transplantation in patients with primary sclerosing cholangitis or autoimmune liver diseases. Hoffmann K, et al Langenbecks Arch Surg. 2014 Dec; 399(8):1001–9

Revised Mayo Risk Score: A revised natural history model for primary sclerosing cholangitis. Kim WR, et al Mayo Clin Proc. 2000 Jul;75(7):688–94

Fib 4: Value of Liver Function Tests in Cirrhosis. Sharma P. J Clin Exp Hepatol. 2022 May–Jun;12(3): 948–964

APRI score: Aspartate Aminotransferase-To-Platelet Ratio Index: a Potential Predictor of Prognosis in the Most Common Types of Cirrhosis. Khajehahmadi Z, et al Clin Lab. 2021 Nov 1; 67(11)

ALBI score: Prediction of Transplant-Free Survival through Albumin-Bilirubin Score in Primary Biliary Cholangitis. Fujita K, et al J Clin Med. 2019 Aug 19;8(8):1258

Short-and long-term UK-PSC Risk Scores: Factors Associated With Outcomes of Patients With Primary Sclerosing Cholangitis and Development and Validation of a Risk Scoring System. Goode EC, et al Hepatology. 2019 May;69(5):2120–2135

Raw PI-AOM score: Validation, clinical utility and limitations of the Amsterdam-Oxford model for primary sclerosing cholangitis. Goet JC, et.al Hepatol. 2019 Nov;71(5):992–999

The values that are bolded are the ones that have *p*<0.05, i.e., are statistically significant

### Disease severity classification

PSC severity and expected prognosis were based upon previously validated scores, including the RMRS, Fib-4, APRI, ALBI, Short-Term UK-PSC risk score, and PI-AOM for PSC [23–26] (Table 2). We classified these continuous scores into risk-based categorical groups (Table 1S). Binary assessment of T2-MRCP recorded the presence of DS (i.e., yes or no), HGS (i.e., yes ≥ 75% or no < 75%), and, on T1-MRC, the presence of PFS or NFS.

### Definition of clinical events

Patients entered the survival analyses at the time of GA-MRI. In March 2022, patient records were censored at date last seen, if there were no adverse events. We recorded survival status (alive, deceased, OLT) and date and type of liver-related events, which included OLT and liver-related death (i.e., primary endpoints) versus ERCP-guided dilatation and/or stenting; variceal bleeding, encephalopathy, and/or ascites (all signs of hepatic decompensation); and the new occurrence of cholangio-, gallbladder, or hepatocellular carcinoma (i.e., secondary endpoints) (Table 1).

### MRI exam protocol

All examinations were performed on a 3-Tesla MR (MAGNETOM Trio Tim, Siemens). T2-weighted MRCP was performed according to Hoeffel et al’s protocol and adhered to International PSC Study Group recommendations [27, 28]. MRCP images included a respiratory-triggered, 3D, heavily T2-weighted sequence in the coronal plane and a breath-hold, thick-slab, single-shot, 2D, heavily T2-weighted sequence in the coronal and oblique coronal projections. Axial and coronal T1-MRC images were obtained during breath-holding, 20 min after intravenous injection of 0.025 mmol/kg body weight gadoxetic of acid at 1 mL/s, followed by a 20-mL saline bolus. The examination parameters for the whole MRI are given in Table 2S.

### Image analysis

MRI exams were anonymized. Then, the MR images were independently evaluated on a commercially available workstation (PACS system) by six radiologists blinded to clinical data. The readers’ (R) level of experience in abdominal imaging were as follows: R1, 3 years; R2, 4 years; R3, 5 years; R4 and R5, 10 years; and R6, > 20 years. R1, R2, and R3 were considered novices (i.e., residents); and R4, R5, and R6, experts. To test intra-reader agreement, R6 reviewed the images twice, 12 weeks apart. Before analyzing the study cohorts, the radiologists reviewed 20 non-study liver GA-MRIs jointly to set the diagnostic criteria for impaired excretion (PFS) vs. normal excretion (NFS) and standardize their reporting of DS and HGS on T2-MRCP. Then, study images were assessed in two different sessions: first, conventional T2-weighted-3D-MRCP and single-shot 2D-MRCP; second, the entire GA-MRI, including DWI, dynamic, and T1-MRC images.

**Fig. 2:**
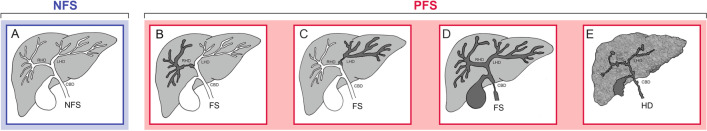
Scheme demonstrates (**A**) normal biliary contrast excretion (white) in a PSC patient with no functional stricture (NFS). No contrast opacification (dark gray) is seen in a functional stricture (FS) in (**B**) the right hepatic duct, (**C**) the left hepatic duct, and (**D**) the hilum. **E** Hepatocellular dysfunction (HD) in very advanced PSC with cirrhosis as noted by the pruned tree appearance of the bile ducts and the shrunken nodular rim of the liver

The readers graded intra- and/or extrahepatic bile duct changes on T2-MRCP sequences according to various guidelines, recording the presence of DS or HGS [5, 24, 29] (Table 3). On 20-min T1-MRC images, patients were stratified into two groups: normal contrast excretion at 20 min (NFS) or impaired excretion (PFS), i.e., no contrast seen either to first-order (LHD or RHD) bile ducts, or CHD/hilum, or CBD or none at all at 20 min (Fig. 2). Then, using multiple-choice format (i.e., RHD, LHD, hilum or CBD), readers were asked to select the location of FS on T1-MRC, and of DS or HGS on T2-MRCP. Stricture location on T1-MRC was determined by the site where contrast media stopped, whereas on T2-MRCP stricture site was assigned to the smallest-diameter segment of the bile duct. Next, they were asked to rate their degree of confidence in stricture location using the Likert scale, where 1 = not confident at all, 2 = slightly confident, 3 = somewhat confident, 4 = fairly confident, and 5 = completely certain.

**Table 3:** Imaging criteria for dominant stricture (*DS*), high-grade stricture (*HGS*), and potential functional stricture (*PFS*) of bile ducts and MR features for hepatocellular dysfunction (*HD*)

MRI technique	Nomenclature	Definition of stricture
T2-MRCP based on ERCP-Criteria	**Dominant stricture**	A biliary stenosis found on ERCP with a diameter of ≤ 1.5 mm in the common bile duct or of ≤ 1 mm in the hepatic duct within 2 cm of the hilum
T2-MRCP	**High-grade stricture** [8]	A biliary stenosis found on MRI/MRCP with ≥ 75% reduction in the common bile duct or hepatic duct lumen with pre-stenotic dilatation
T1-MRC	**Potential functional stricture (PFS)**	No CM excretion into the main RHD or LHD within 2 cm of the hilum or CHD or CBD, 20 min after injection of gadoxetic acid in the absence of cirrhosis (Fig. 2)
Stratification on T1-MRC	Score	Description
No functional stricture (NFS)	0	CM excretion into the RHD or LHD or distal CBD within 20 min, i.e., HBP
Potential functional stricture (PFS)	1	No CM excretion into the main RHD or LHD within 2 cm of the hilum or CHD or CBD, 20 min after injection of gadoxetic acid
Location of stricture	Presence of functional stricture	Definition of the region
CBD	Yes/No	CBD defined as portion of the common hepatic duct from the cystic duct insertion to the ampulla
CHD/hilum	Yes/No	Common hepatic duct defined as from the insertion of the cystic duct to the bifurcation at the hilum
RHD or LHD	Yes/No	RHD and LHD defined as from the center of the hilum to the point where the first intrahepatic duct branches
MRI technique	Appearance of bile ducts	Description of intrahepatic and extrahepatic ducts [8, 30]
T2-MRCP [8, 30]	**Normal**	No visible abnormalities
	**Slight to moderate**	Slight irregularities of duct contour, no stricture, or multiple caliber changes, minimal dilatation
	**Advanced to severe**	Multiple strictures, saccular dilatations almost entire length of duct, severe pruning or diverticulum-like outpouching
Hepatocellular dysfunction		
FLIS on HBP [18]	Score	Description
Liver enhancement	0/1/2	Absent/equal/greater than kidney
Biliary excretion	0/1/2	Absent/IHD/both IHD and EHD
Portal venous sign (PVS)	2/1/0	Absent/equal/greater than liver
Liver dysmorphy [26]	Score	Description
Absent	No	Significant atrophy of one or both lobes and/or marked lobulations of liver surface and/or increase of the caudate/right lobe ratio
Present	Yes	
Portal hypertension		
Splenomegaly [26, 31]	Score	Description [32]
Absent	No	Splenic volume < 381.1 cm ^3^
Present	Yes	Splenic volume ≥ 381.1 cm ^3^
Portosystemic shunts [31]	Score	Description
Absent	No	Portosystemic shunts
Present	Yes	

The values that are bolded are the ones that have *p*<0.05, i.e., are statistically significant

ERCP was performed in PFS patients, if also clinically indicated. When there was true FS, ERCP confirmed and treated it. If no stricture was found, then impaired excretion was presumed to be due to hepatocellular dysfunction (HD) as a result of advanced PSC. In patients with NFS, if there was no clinical indication for ERCP, then follow-up MRI in 6 to 12 months was used to show interim stability without intervention, implying that there was no FS.

A priori, any/all of the following features suggested HD: (a) pruned tree appearance (i.e., peripheral obliteration or reduced arborization of the intrahepatic bile ducts) on T2-MRCP [17]; (b) poor function liver imaging score (FLIS = 0–3) on HBP, i.e., markedly decreased or absent GA liver uptake relative to kidney uptake on the T1-MRC with absent excretion and contrast retention in the portal vein in the HBP, after excluding significant stricture that would cause bile duct dilatation [15, 18, 19]; (c) hepatic dysmorphy [9]; (d) increased liver stiffness on MR-elastography (MRE), if available [33]; (e) enlarged estimated splenic volume, calculated as [mL^3^] = 30 + 0.58 × *L* × *D* × *T* [34]; and (f) portosystemic shunts (Table 3). A cutoff value of 381.1 cm^3^ was chosen to differentiate normal-sized from enlarged spleens (Table 1S) [32, 35].

**Fig. 3:**
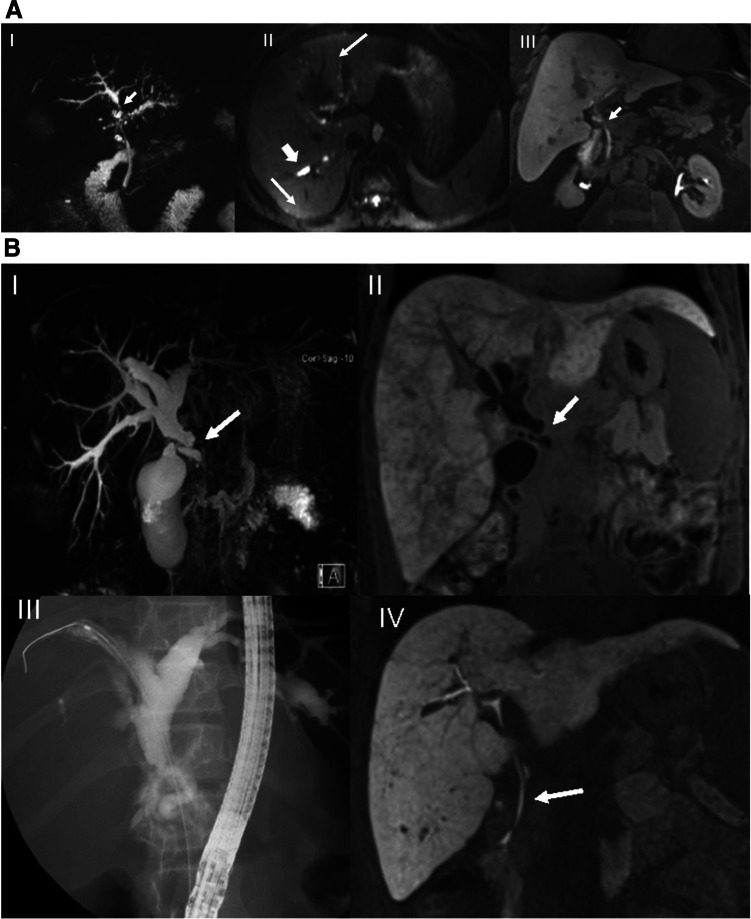

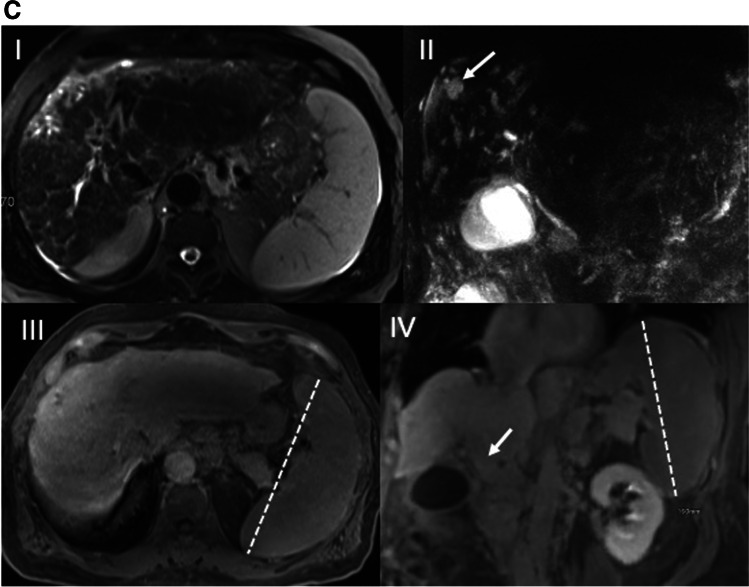
**A **A 56-year-old male with known PSC for four years. Conventional T2-MRCP (I) is suspicious for a high-grade stricture (HGS) in the hilum (arrow). Furthermore, moderate to significant dilatations and irregularities of the IHD are seen. Axial DWI (II) shows dilatation of the intrahepatic bile ducts in the right liver lobe (short thick arrow), as well as associated high SI in both lobes consistent with segmental cholangitis (thin arrows). Twenty-minute coronal HBP image (III) shows timely contrast media excretion via CBD into the duodenum (arrow), excluding a functional stricture. Thus, this was a false-positive MRCP diagnosis of DS and HGS. The patient developed no adverse event(s). **B** A 20-year-old female with PSC and a 2-year history of Crohn’s disease. Coronal MRCP (I) shows a suspected significant hilar stricture (arrow) with distinct dilatation of the intrahepatic bile ducts. On the 20-min coronal hepatobiliary phase (II), no contrast media excretion was seen in the biliary system compatible with a functional stricture (FS). There was also heterogeneous liver parenchymal enhancement. ERCP (III) 1 week later confirmed the diagnosis of FS in the mid-CBD, which was treated with dilatation. Three-month follow-up MRCP (IV) showed timely biliary excretion 20 min after contrast administration, as well as more homogeneous liver parenchymal enhancement, indicative of improved function after removal of the FS. **C** A 62-year-old male with PSC for 23 years. Axial T2-TSE (I), coronal 3D-MRCP (II), and axial (III) and coronal (IV) T1 3D GRE in the HBP show signs of advanced PSC on MRCP with cystic dilatation (II, arrow), and multiple stenoses of the intrahepatic bile ducts, as well as a pruned tree appearance (I, II). The liver shows diminished uptake of hepatobiliary contrast agent relative to the left kidney (III, IV) without excretion at 20 min (IV, arrow), indicative of hepatocellular dysfunction (HD) with signs of cirrhosis, including dysmorphy and portal hypertension (including splenomegaly, III and IV). Four months later, the patient underwent OLT

Finally, we checked for the presence of a secondary malignancy, such as CCA, gallbladder cancer, or HCC. Examples that compare the findings on T1-MRC vs. T2-MRCP are shown in Fig. 3A-C.

### Statistical analysis

Metric data are presented as means ± standard deviations or median and quartile, depending upon distribution. Nominal data are presented as absolute frequencies and percentages. Differences in variables between the NFS and PFS groups were analyzed using Student’s *t*-test, with Welch correction in case of unequal variances, or the Wilcoxon rank-sum test, as appropriate. Categorical data were evaluated by the Chi-squared test or Fisher’s exact test. Inter- and intrareader agreements between radiologists were assessed using Fleiss’ and Cohen’s kappa, respectively. The Likert scale evaluated reader confidence regarding stricture location. Event-free survival time was defined as the time interval from MR diagnosis to the first occurring liver-related event, as defined above. Survival analysis was performed using Kaplan–Meier curves, a Cox proportional hazard model, and 95% confidence intervals (CI).

The level for statistical significance was set at *p* < 0.05. Statistical analyses were performed using R Studio (Version 1.4.1717) and IBM SPSS (version 26).

## Results

### Cohort characteristics

We included 129 patients, 52F/77 M, mean age 41.4 ± 14.7 years (range, 18.3–77.6 years), diagnosed with PSC according to society guideline MRCP features [28, 36]. Of these, 44 (33.9%) had biopsy-confirmed PSC. Eighty-two patients had histologically verified IBD: 49 with ulcerative colitis; and 33 with Crohn’s disease (Table 1). Mean and median follow-up post-MRI were 43.5 months and 30.7 months, respectively. Gender, age, and BMI did not differ significantly between PFS vs. NFS patients (all *p* > 0.05).

By March 1, 2022, seven (5.4%) and 19 (14.7%) of the 129 patients had died or received OLT, respectively. Other recorded events included ERCP-guided treatment in 36 (27.9%); variceal bleeding in six (4.7%); encephalopathy in two (1.6%); HCC development in two (1.6%); and CCA or gallbladder cancer in six (4.7%). Overall, 55 patients (42.3%) had at least one event during the observation period (Table 1).

### MRI results

#### Inter- and intrareader agreement

On 20-min T1-MRC, inter-reader agreement among the six radiologists and for the three residents for NFS vs. PFS was substantial (*κ* = 0.76 and* κ* = 0.70, respectively), and almost perfect between the three experts (*κ* = 0.83). The intra-reader agreement (R6) was also substantial (*κ* = 0.79). However, there was fair to poor agreement for DS (*κ* = 0.22 all and *κ* = 0.08 experts) and moderate agreement for HGS (*κ* = 0.58 all and* κ* = 0.52 experts) on T2-MRCP. For FS location, inter-reader agreement was significantly higher on T1-MRC than on T2-MRCP (*κ* = 0.68 vs. *κ* = 0.14, respectively) (Table 3S).

Overall, 87 (67%) patients had normal excretion on GA-MRI, i.e., NFS, and 42 (33%) patients had impaired 20-min excretion on GA-MRI, i.e., PFS (Table 1). Based on IPSCSG criteria [8], all subsequent results are provided only for R6, the most experienced radiologist, as inter-reader agreement for experts was almost perfect. Additionally, below is the data for the 3 diagnostic algorithms (PFS, HGS, DS) for the median of the six readers.

Excluding the 4 tied cases, median PFS diagnosis was PFS in 39 cases (31%) and NFS in 86 cases (69%) for all readers, whereas our expert reader diagnosed PFS in 42 cases (33%) and 87 cases (67%). Median HGS diagnosis was no HGS in 65 cases (50%) and HGS in 64 cases (50%), whereas our expert reader diagnosed no HGS in 87 cases (67%) and HGS in 42 cases (33%). Excluding the 34 tied cases, median DS diagnosis was no DS in 53 cases (56%) and DS in 42 cases (44%) whereas our expert diagnosed no DS in 83 cases (64%) and DS in 46 cases (36%). Using the median T1-MRC diagnoses, differences between PFS and NFS were still significant for events, for example OLT (*p* < 0.001, PFS *n* = 14/39, NFS *n* = 5/90) and therapeutic ERCP (*p* < 0.001, PFS *n* = 36/39, NFS *n* = 0/90) (4 ties were counted as NFS).

### Reader selection of/ confidence in stricture location

FS location on T1-MRC was as follows: 16 in the hilum/CHD, 18 in CBD, 3 in LHD, and none in RHD for the 37 patients with proven strictures (36 by ERCP, one by histology). The Likert scale average, expressing reader diagnostic confidence, on T1-MRC versus T2-MRCP, was 4.5 versus 3.1 for all six readers, 4.9 versus 3.0 for the three experts, and 4.1 versus 3.2 for the three residents. Furthermore, on T1-MRC, the six readers stated they lacked confidence on 6 cases average, which decreased to 0.3 cases for the three experts. For T2-MRCP, on average, the six readers stated they were not confident on 37 cases versus 30 cases for the experts for DS, and on 32 versus 29 cases for HGS.

### Image quality of T1-MRC and 3D-MRCP

With regard to T1-MRC, none of the six readers noted artifacts. All readers independently rated the quality of 3D-MRCP according to the IPSCSG criteria [8]. Because there was no statistically significant difference in the mean artifact score between expert reader R6 and all readers, all results are for R6. On 3D-T2-MRCP, average readers vs. R6 reported 107 and 108 cases with no artifacts and 22 and 21 cases, with minor artifacts, respectively, none of which affected diagnostic performance. With regard to blurring, both average readers and R6 found minor blurring in 28 cases, but diagnostic performance was unaffected. Biliary tree delineation for average readers vs. R6 was excellent in 59.8 and 62 cases, good in 40.2 and 38 cases, fair in 19.7 and 21 cases, and poor in 9.3 and 8 cases, respectively.

## Spleen volumetrics

Spleen volumes were significantly higher (*p* = 0.016) with impaired excretion (PFS), and significantly more patients with splenomegaly had liver-related events (*p* < 0.001) (Table 2 and Table 1S).

### Magnetic resonance elastography (MRE) values

Only 34 (26.4%) patients underwent MRE, 12 with PFS and 22 with NFS. Differences in kPa between PFS and NFS were not significant (*p* = 0.301), although mean values for PFS were higher (3.3 ± 1.50 kPa vs. 2.8 ± 0.50 kPa). Similarly, kPa values were not significantly different (*p* = 0.221) regarding clinical events vs. no events. However, mean MRE values were again higher in those who experienced events (3.3 ± 1.44 kPa vs. 2.8 ± 0.48 kPa).

### ERCP and ERCP-guided treatments

**Fig. 4:**
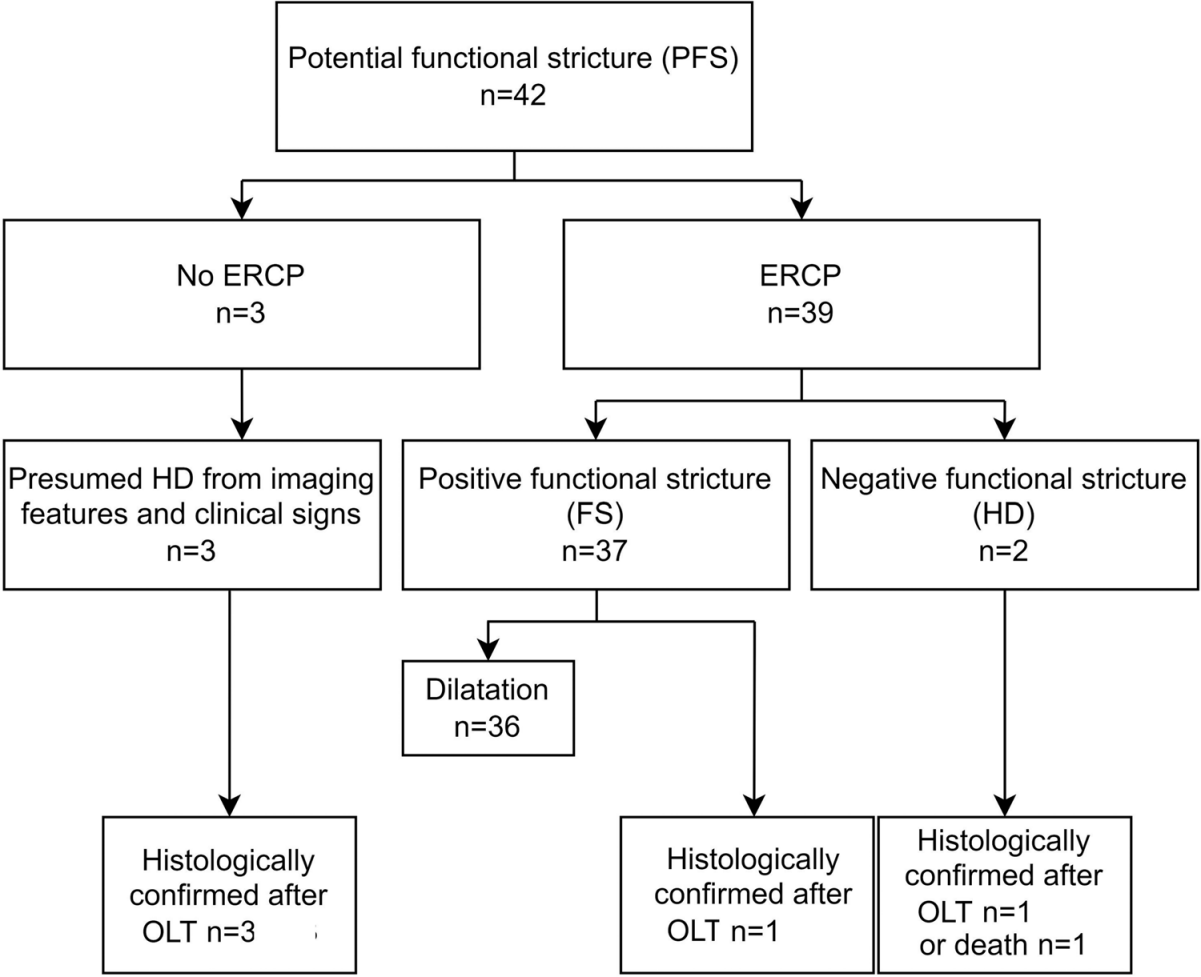
Flowchart shows ERCPs, the gold standard, performed in patients with a potential functional stricture. Histopathologic confirmation of HD was obtained in five patients. As well, the one patient who was suspected of having FS, but had no ERCP, had FS confirmation after OLT

Forty-four (34.1%) ERCPs were performed, 39 in PFS (92.9%) and five in NFS (5.7%) patients due to clinical suspicion. FS was confirmed within 1.7 months (mean) post-MRI in 37 of 39 PFS patients. Thirty-six patients had therapeutic ERCP; the last patient underwent OLT 5 months later. Two PFS patients had no stricture on ERCP and were presumed to have HD. This was confirmed within 6 months; at post-mortem in one, and at OLT in the other. Regarding the three PFS (7.1%) patients who did not have ERCP, all had histologically confirmed HD, but no FS at OLT (Fig. 4).

Sensitivity, specificity, and accuracy for therapeutic ERCP done within 3 months of MRI were 100%, 89%, and 91%, respectively for T1-MRC, but only 81%, 41%, and 50% for T2-MRCP DS diagnosis, and 58%, 76%, and 71% for T2-MRCP HGS diagnosis. The negative predictive value of NFS diagnosis on T1-MRC was 100% (Table 4S).

### Laboratory tests and clinical scores

Lab tests and established clinical scores between normal (NFS) vs. impaired (PFS) excretion patients demonstrated a statistically significant difference for most parameters, including the Revised Mayo, ALBI, and Short-Term UK PSC risk scores (all *p* < 0.001), as well as APRI (*p* = 0.015), FIB-4 (*p* = 0.031), and AOM (*p* = 0.001) (Table 2).

### Correlation with clinical events and endpoints

Table 1S illustrates the frequency of clinical events based upon T1-MRC-diagnosis, T2-MRCP-diagnosis, splenic volume, and clinical scores.

Whereas all PFS patients (100%) had at least one event, only 15%, i.e., circa one of seven NFS patients, experienced an event (Table 1). Significantly more events were recorded when HGS (≥ 75%) (67% vs. 31.0%, *p* < 0.001) or DS (51%, vs. 28%, *p* = 0.014) was diagnosed on T2-MRCP or when splenomegaly (SV > 381.1 cm^3^) was present (*p* < 0.001). Likewise, there were more events in patients with MR-derived, high-risk clinical scores (*p* < 0.001).

Nine NFS patients reached a primary endpoint (10%), including 4/87 (5%) who died and 5/87 (6%) who received OLT. Seventeen of 42 PFS patients reached a primary endpoint (41%, *p* < 0.001), including 3/42 (7%) who died and 14/42 (33%) who underwent OLT.

Two cases of encephalopathy occurred in PFS (5%) patients, while there were three variceal bleeds in each group, NFS (3%) and PFS (7%). Finally, malignancy occurred in three PFS (7%) and five NFS (6%) patients.

**Fig. 5:**
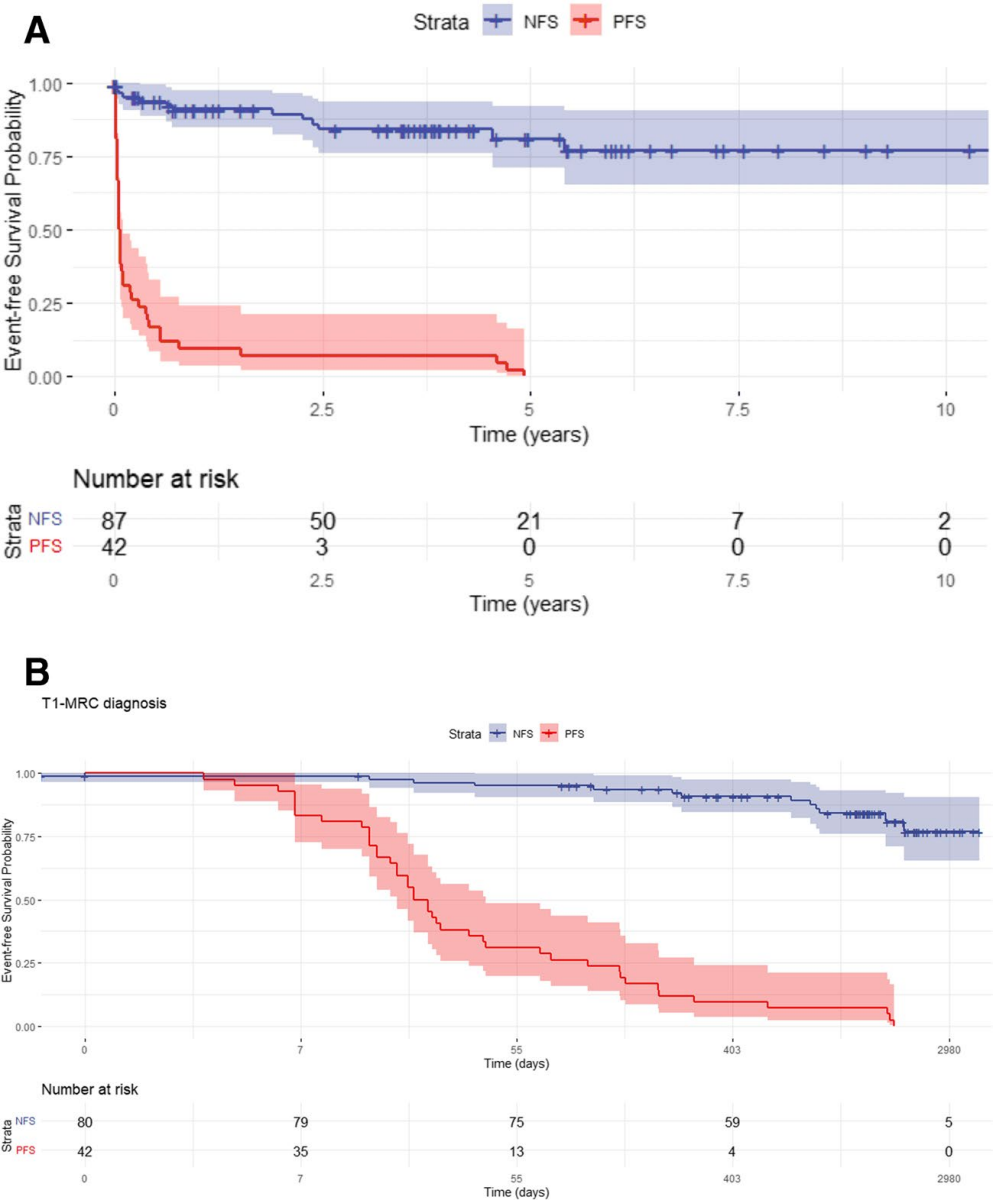
Kaplan–Meier curves **(A)** show estimated event-free survival time in the NFS group: 8.32 years mean (7.204 – 9.425 years 95% confidence interval) and the PFS group: 1.11 years median (0.0–2.600 years 95% confidence interval) with a mean of 2.97 years (1.50–4.45 years 95% confidence interval). A log rank test (Mantel-Cox) was significant with *p* < 0.001. **B** After log conversion, we could better see the spread of events during the first year post-MRI

The Kaplan–Meier curve for event-free patient survival showed a markedly higher probability of event occurrence over time with PFS vs. the normal-excretion patients. The difference was especially visible in the first year post-MRI, which was better seen after log-conversion of the x-axis (*p* < 0.001) (Fig. 5A, B).

Univariate Cox regression showed that the risk of event occurrence, including death and OLT, rises ~ 19-fold annually for PFS patients [95% CI, 9.9–38.2]. However, HR was only 1.9 for HGS [95% CI, 1.4–2.6] and 2.1 for DS [95% CI, 1.2–4.0] based on T2-MRCP diagnosis. Clinical scores were included in the multivariate analysis to achieve the best fit. The PFS and AOM were identified as independent risk factors with HRs of 28.6 [95% CI, 12.5–65.5] and 1.6 [95% CI, 1.0 – 2.5], respectively (Table 4).

**Table 4:** Cox regression univariate and multivariate analysis of MRI features, clinical scores, and laboratory tests for all events

Patient characteristics, *n* = 129	Univariate analysis A			Multivariate analysis B		
	HR	95%CI	*p* value	HR	95%CI	*p* value
T1-MRC, NFS (no) vs. PFS (yes)	19.5	9.9–38.2	** < 0.001**	28.6	12.5–65.5	** < 0.001**
T2-MRCP high-grade stricture < 75% (no), ≥ 75% (yes)	1.9	1.4–2.6	** < 0.001**			
T2-MRCP dominant stricture (no), or (yes)	2.1	1.2–4.0	**0.016**			
Spleen volume, by increments of 100 cm^3^	1.0	1.0–1.0	** < 0.001**			
MR elastography	1.6	1.0–2.7	0.056			
Age, step size 10 years	1.0	1.0 – 1.0	0.142			
Gender, female (0) vs. male (1)	1.3	0.8–2.3	0.310			
BMI	1.0	0.9–1.1	0.831			
MELD, per point	1.1	1.0–1.1	**0.002**			
Revised Mayo Risk Score	1.5	1.3–1.8	** < 0.001**	0.8	0.5–1.2	0.199
FIB-4 index	1.1	1.0–1.2	**0.008**			
APRI	1.1	1.0 – 1.2	0.154			
ALBI	2.4	1.7–3.3	** < 0.001**			
Short‐Term UK‐PSC Risk Score	1.8	1.5–2.3	** < 0.001**	0.9	0.5–1.7	0.770
Amsterdam Oxford Model	1.7	1.3–2.2	** < 0.001**	1.6	1.0–2.5	**0.037**
Bilirubin, per mg × dl^−1^	1.0	1.0 – 1.1	**0.002**			
Albumin, by increments of 5 g/ml	0.7	0.6–0.8	** < 0.001**			

Multivariate analysis for best fit (determined by *R*^2^, which was 0.835) is given

*n*, number of the patients; *HR*, hazard ratio; *CI*, confidence interval; *BMI*, body mass index; *NFS*, no functional stricture; *PFS*, potential functional stricture; *MELD*, model of end-stage liver disease; *FIB-4*, Fibrosis-4 Index; *APRI*, AST to Platelet Ratio Index; *ALBI*, albumin-bilirubin grade

The values that are bolded are the ones that have *p* < 0.05, i.e., are statistically significant

## Discussion

Our suggested binary stratification into normal (NFS) versus impaired excretion (PFS) on 20-min hepatobiliary phase (T1-MRC) images had 100% positive predictive value. In other words, all 42 PFS patients had either a functional stricture or hepatic dysfunction. ERCP confirmed all but one functional stricture which was later confirmed histologically at OLT. Similarly, the diagnosis of NFS had a 100% negative predictive value. Although the experts had higher inter-reader agreement than the residents, i.e., almost perfect vs substantial, on T1-MRC, this was far better than T2-MRCP agreement for DS and HGS, which had only poor and moderate agreement, respectively. Even using the median T1-MRC diagnoses of all 6 readers, differences between PFS and NFS were still significant for events. Further confirmation of the robustness of PFS over DS and HGS is the relatively few cases with discrepant diagnoses between residents and experts when interpreting T1-MRC (3%) versus T2-MRCP (16–26%).

NFS versus PFS diagnosis correlated far better with clinical outcomes (HR 19.5 vs 2.1 and 1.9) than DS or HGS diagnosis on conventional T2-MRCP, and there was far better agreement on stricture location. Because clinically significant strictures are so common, early and accurate stricture diagnosis is imperative. With prompt dilatation and/or short-term stenting, liver failure and OLT can be postponed [37, 38].

To the best of our knowledge, this is the first MR imaging study based upon Erlinger’s 1985 concept of bile physiology. However, in our definition of PFS, we consider the right and left hepatobiliary tree as two separate systems. Therefore, Erlinger’s definition, i.e., bile’s failure to reach the duodenum, only applies to PFS when a significant stricture, i.e., FS, occurs in the hilum or CBD, or in the setting of hepatocellular dysfunction (HD) [39]. However, in PFS, if there is a blockage of the right hepatic duct causing failure of the right liver lobe, bile (i.e., contrast agent) would still reach the duodenum via the patent left hepatic duct, and vice versa. Determining whether excretion on the HBP of GA-MRI, like bile flow, is normal versus impaired in the presence of significant bile duct narrowing has improved both sensitivity and reader confidence in the diagnosis and location of biliary obstruction [13].

We compared the inter-observer variability between DS and HGS based on T2-MRCP, and our proposed potential functional stricture (PFS) diagnosis on T1-MRC, finding that T1-MRC diagnosis is clearly superior to all T2-MRCP–based scores. We found only moderate and fair agreement for T2-MRCP–based HGS and DS, respectively. T2-MRCP’s poor performance, even among PSC experts, is likely due to the subjectivity in determining bile duct stricture severity [7]. Moreover, applying ERCP criteria to T2-MRCP led to poor anatomic depiction, lack of functional information, and/or an underdistended biliary tree on T2-MRCP contributing to both false-positive and false-negative stricture misdiagnoses [7, 25, 40]. In contrast, there was almost perfect inter-reader agreement among experienced readers, and substantial agreement, even among residents, for both PFS presence and location on T1-MRC.

Beyond easy and accurate stricture diagnosis on T1-MRC, we found a statistically significant correlation with well-established prognostic biomarkers, including the RMRS, ALBI, Short-Term UK-PSC risk score, AOM for PSC score, and, most importantly, primary and secondary clinical endpoints. Our results are in line with previous studies where impaired excretion correlated with elevated liver function tests, Mayo PSC risk scores, and downstream biliary obstruction [41, 42]. Spleen volume also correlated significantly with PSC adverse events, differentiating patients with normal versus impaired excretion. As in other chronic liver diseases, splenomegaly [35] and imaging signs of portal hypertension herald advanced PSC, and increased risk of further event(s) [43].

The Kaplan–Meier curves showed a clear separation between adverse clinical events in T1-MRC NFS vs. PFS subpopulations. Furthermore, multivariate analyses using Cox regression showed that T1-MRC-based diagnosis was an independent risk factor for adverse events in PSC patients, outperforming lab tests and clinical scores, even after categorization into an ordinal system. In addition, except for T1-MRC, no other MR metric correlated with established clinical scores, which was unsurprising, as we know T2-MRCP-DS/HGS-diagnosed patients range from asymptomatic to having abnormal liver function tests and/or cholestasis [4].

The limitation of T1-MRC was its inability to separate impaired excretion patients (PFS) with true stricture from those with HD due to end-stage liver disease, i.e., progressive bile duct obliteration and parenchymal fibrosis. However, T2-MRCP findings in the five PFS patients with histologically confirmed HD of advanced PSC showed the classic pruned tree appearance of the intrahepatic bile ducts. Therefore, the combined features of T1-MRC, T2-MRCP, and all other MR images vastly improved the distinction between FS and/or HD [15], as has previously been described [15, 16].

In equivocal clinical cases, a confident diagnosis on T1-MRC can sway the decision whether or not to perform ERCP. In this sense, T1-MRC can help triage patients for ERCP. In routine clinical practice, the management of PFS, based on a multiparametric MR exam, should include interdisciplinary discussion between experienced radiologists and gastroenterologists to reduce the burden of unnecessary ERCPs, as recommended by the IPSCSG [8].

Moreover, the superiority of multiparametric GA-MRI, providing simultaneous anatomic plus global/segmental functional hepatobiliary information, using functional liver imaging score (FLIS), can show PSC’s progression to end-stage cirrhosis with portal hypertension [11, 18–20].

Importantly, in our study, we did not detect any CCA in the PFS diagnosis. The two (1.55%) confirmed cases of CCA in our cohort occurred in the NFS group. Our results are similar to those of Villard et al where very few of their cohort, i.e., 2%, had CCA and none in a significant stricture [44]. The real burden of CCA in a DS or FS should be further evaluated in a prospective multicenter study.

We acknowledge the inherent limitations of our retrospective study. First, by considering ERCP not only a secondary event, but also as the gold standard, our study had an indication bias. However, our main goal was to see if T1-MRC could yield an accurate and confident imaging diagnosis with better agreement among radiologists, i.e., a less subjective method than measurements (e.g., DS) or approximations (e.g., HGS). Therefore, we had to use ERCP to confirm our diagnoses which then led to either dilatation and/or stenting, if FS was diagnosed. This may have caused an overestimation of PFS events. Therefore, we advise caution in interpreting our prognostic results. Secondly, we must acknowledge that we used the term DS to interpret T2-MRCP images, despite the MR Working Group of the IPSCSG’s recent recommendation that the term DS should not be used with MRCP [8]. Thirdly, regarding our primary endpoints, we had almost 15% of our cohort who either underwent OLT or had liver-related mortality. We believe this relatively high percent of primary adverse events within a short follow-up time is because our tertiary care patients were severely ill compared to the average PSC patient. This is a well-known phenomenon in tertiary centers [45]. Fourthly, our retrospective study design, by skewing our inclusion cohort, caused rather short mean and median follow-up times (43.5 months and 30.7 months, respectively) as compared to our long observation period. Fifthly, we gave the results based on the analysis of the most experienced reader (almost perfect) according to the IPSCSG criteria [8] which may have led to an overestimation; however, the difference between experts and residents is negligible, as the median values for all readers show. Additionally, the median values also correlated with the events. Evenso, we advise caution in interpreting our prognostic results. Sixthly, all but three patients in the impaired excretion group underwent ERCP, due to suspected end-stage PSC, which was histologically confirmed at OLT or post mortem. Not all patients underwent ERCP because of its invasive nature. Our physicians only perform ERCP when clearly clinically indicated to avoid complications. However, no functional stricture (NFS) patient had subsequent loss of liver function or any event 6 to 12 months after MRI. Finally, liver stiffness for PSC severity prediction was measured with MR elastography in only one-third of our cohort. Our results showed no statistically significant difference in liver stiffness between NFS and PFS patients nor between events and no-events, likely due to the relatively small group [33]. Even so, MR elastography cannot diagnose FS.

In conclusion, T1-MRC allows a more confident and reproducible diagnosis and localization of stricture than T2-MRCP. PFS correlated better than DS or HGS with lab values, clinical scores, and outcomes.

### Supplementary Information

Below is the link to the electronic supplementary material.

Supplementary file1 (PDF 203 kb)

## Abbreviations

ALBI: Albumin-bilirubin score
AOM: Amsterdam-Oxford model for PSC
CBD: Common bile duct
CCA: Cholangiocarcinoma
DS: Dominant stricture
ERCP: Endoscopic retrograde cholangiopancreatography
Fib-4: Fibrosis-4-index
GA-MRI: Gadoxetic acid-enhanced MRI
HBP: Hepatobiliary phase
HD: Hepatocellular dysfunction
HGS: High-grade stricture
IBD: Inflammatory bowel disease
IPSCSG: International PSC study group
LHD: Left hepatic duct
MRI: Magnetic resonance imaging
NFS: No functional stricture
OLT: Orthotopic liver transplantation
PFS: Potential functional stricture
PSC: Primary sclerosing cholangitis
RHD: Right hepatic duct
T1-MRC: T1-weighted hepatobiliary phase-enhanced MR cholangiography
T2-MRCP: T2-weighted magnetic resonance cholangiopancreatography

## Declarations

### Guarantor

The scientific guarantor of this publication is Dr. Ahmed Ba-Ssalamah.

### Statistics and biometry

Dr. Michael Weber kindly provided statistical advice for this manuscript.

### Some study subjects or cohorts overlap

No study subjects or cohorts overlap.

### Methodology

• retrospective

• diagnostic or prognostic study

• performed at one institution
