# Supplementary material for: Diagnosis of functional strictures in patients with primary sclerosing cholangitis using hepatobiliary contrast-enhanced MRI: a proof-of-concept study
Source: Eur Radiol. 2023 Jul 20;33(12):9022–37. doi: 10.1007/s00330-023-09915-3 (PMC10667158; doi:10.1007/s00330-023-09915-3)
Supplement: Supplementary file 1 — Supplementary file1 (PDF 203 kb) [file 330_2023_9915_MOESM1_ESM.pdf]

Diagnosis of Functional Strictures in Patients with Primary Sclerosing Cholangitis Using Hepatobiliary Contrast-enhanced MRI: A Proof-of-Concept Study

ELECTRONIC SUPPLEMENTARY MATERIAL

Table 1S: Association between T1-MRC, and T2-MRCP diagnosis, clinical scores and adverse events.

Imaging Modality	Scoring method	No Event	Event	Comparison	p-value*
**T1-MRC**	No functional stricture (NFS)	74 (85.1%)	13 (14.9%)	NFS vs. PFS	**<0.001**
	Potential functional stricture (PFS)	0 (0%)	42 (100%)		
**T2-MRCP**	No dominant stricture (NDS)	33 (70.7%)	13 (28.3%)	No vs. Dominant Stricture	**0.021**
	Dominant stricture (DS)	41 (49.4%)	42 (50.6%)		
**T2-MRCP**	No High-grade Stricture (NHGS)	60 (69.0%)	27 (31.0%)	No High-grade stricture < 75% vs. high-grade stricture ≥ 75%)	**<0.001**
	HGS	14 (33.3%)	28 (66.7%)		
**Spleen volume[1]**	Normal (≤ 381.1 cm³)	58 (71.6%)	23 (28.4%)	Normal vs. splenomegaly	**<0.001**
	Splenomegaly (>381.1 cm³)	14 (31.1%)	31 (68.9%)		
**Revised Mayo Risk Score (RMRS)**	Low risk	62 (77.5%)	18 (22.5%)	Low vs. intermediate and high risk	**<0.001**
	Intermediate risk	12 (32.4%)	25 (67.6%)		
	High risk	0 (0%)	12 (100%)		
**ALBI Grade**	1	65 (73.0%)	24 (27.0%)	1 vs. 2 and 3	**<0.001**
	2	9 (27.3%)	24 (72.7%)		
	3	0 (0%)	7 (100%)		
**APRI Score**	≤ 1.17	68 (64.8%)	37 (35.2%)	≤ 1.17 vs. > 1.17	**<0.001**
	> 1.17	6 (25.0%)	18 (75.0%)		
**FIB-4 Index**	≤1.3	59 (70.2%)	25 (29.8%)	≤ 1.3 vs. > 1.3	**<0.001**
	>1.3	15 (33.3%)	30 (66.7%)		

**Risk group of Amsterdam-Oxford model[2]**	Low risk	28 (77.8%)	8 (22.2%)	Low to intermediate vs. moderate to high risk	**<0.001**
	Low to intermediate risk	23 (76.6%)	7 (23.3%)		
	Moderate risk	17 (48.6%)	18 (51.4%)		
	High risk	6 (21.4%)	22 (78.6%)		

* Chi-square tests comparing in a 2x2 cross-table.
1 Three patients underwent splenectomy
2 Missing laboratory parameters for one patient

Table 2S MRI protocol

Sequences	Slice orientation	Matrix[1]	Voxel mm	FOV[1] mm	SL[1] mm	gap mm	TR ms	TE ms	FA Degree	Time s
GRE T1 (2D flash) in-phase	Axial	320x320	0.5x0.5x5	350	5	1	130	2.38	70	21
GRE T1 (2D flash) opposed phase	Axial	320x320	0.5x0.5x5	350	5	1	130	4.85	70	21
2D T2-MRCP breath-hold thick slab	Radial	384x384	1.0x1.0x0.45	380	55	0.5	5500	454	180	7x6
3D T2-MRCP respiratory-triggered	Coronal	384x384	0.9x0.9x0.9	350	0.9	0	4500	700	90	228
T1 VIBE Fat Sat unenhanced and gadoxetic-enhanced (arterial, portal-venous, 5 min/transitional)	Axial	512x384	1.1x1.1x2.5	350	2	0.5	2.6	0.92	13	16x3
T1 VIBE portal-venous	Coronal	232x256	2.0x2.0x2.0	500	2	20%	2.6	0.92	13	16
DWI TSE-EP/ADC	Axial	384x288	1.8x1.8x5	350	5	1	5100	67	90	133
T2 HASTE with fatsat	Axial	320x320	1.1x1.1x5	350	5	1	1800	150	150	21x2
T2 HASTE	Coronal	512x512	0.5x0.5x1.2	380	4	0	1200	78	150	228
T1 VIBE Fat Sat 20 Min post contrast (HBP)	Axial	512x384	1.1x1.1x2.5	350	2	0.5	2.6	0.92	13	16
T1 VIBE Fat Sat 20 Min post contrast (HBP)	Coronal	384x384	1.3x1.3x2.2	380	2	0.44	3.59	1.4	20	16
2D SE-EPI MRE	Axial	100x100	1.6x1.6x8	420	8	0.25	1000	45	90	15

[1]Average FOV, matrix and slice thickness as these vary with patient size
VIBE = volume interpolated breath-hold examination
DWI TSE-EP/ADC= diffusion-weighted imaging Turbo spin echo-Echo planar/ Apparent diffusion coefficient
HASTE = The half-Fourier single-shot turbo spin-echo
2D SE-EPI MRE = *2D* echo planar imaging (GRE) and *2D* spin echo imaging

Table 3S: Inter-reader agreement for all readers including experts and novices, i.e., residents (3-5 years) (N=6), below for experts only (N=3) (>10 years) using Fleiss' kappa statistics.

MR features	Fleiss-Kappa	95% CI	Agreement all (n=6) Readers
**T2-MRCP Dominant Stricture**	0.22	0.17 – 0.27	fair
**T2-MRCP High-Grade Stricture**	0.58	0.47 - 0.66	moderate
**T1-MRC NFS vs. PFS on HBP**	0.76	0.71 – 0.80	substantial
**Region of stricture on T2-MRCP** None/RHD/LHD/CHD (hilum) /CBD	0.14	0.12 - 0.16	poor
**Region of stricture on T1-MRC** None/RHD/LHD/CHD (hilum) /CBD	0.68	0.61 - 0.77	substantial
**Bile duct changes on T2-MRCP** None/RHD/LHD/CHD (hilum) /CBD	0.57	0.41 - 0.66	moderate
**Presence of liver cirrhosis** - Yes/No	0.67	0.62 - 0.72	substantial
**Splenomegaly** - Yes/No	0.63	0.52 - 0.74	substantial
**Portosystemic Shunts** - Yes/No	0.71	0.67– 0.79	substantial
**MR features**	**Fleiss-Kappa**	**95% CI**	**Agreement expert (n=3) Readers**
**T2-MRCP Dominant Stricture**	0.08	-0.02 – 0.19	poor
**T2-MRCP High-Grade Stricture**	0.52	0.37 – 0.67	moderate
**T1-MRC NFS vs. PFS on HBP**	0.83	0.72 - 0.94	almost perfect
**Region of stricture on T2-MRCP** None/RHD/LHD/CHD (hilum) /CBD	0.04	0.04 - 0.05	poor
**Region of stricture on HBP** None/RHD/LHD/CHD (hilum) /CBD	0.88	0.61 - 0.91	almost perfect
**Bile duct changes on T2-MRCP** None/RHD/LHD/CHD (hilum) /CBD.	0.69	0.45 - 0.72	substantial
**T1-MRC stratification into: NFS, FS, HD**	0.89	0.81 - 0.97	almost perfect
**Presence of liver cirrhosis** -Yes/No	0.81	0.61 - 0.88	almost perfect
**Splenomegaly** - Yes/No	0.87	0.78 - 0.97	almost perfect
**Portosystemic Shunts** - Yes/No	0.83	0.66 - 0.86	almost perfect

The **kappa statistic**: Values < 0 as indicating poor and 0.00–0.20 as slight, 0.21–0.40 as fair, 0.41– 0.60 as moderate, 0.61–0.80 as substantial, and 0.81–1.00 as almost perfect agreement.
Reader (N) = number of the readers.
CBD=common bile duct, CHD= common hepatic duct, RHD=right hepatic duct, LHD=left hepatic duct, NFS=no functional stricture, PFS=potential functional stricture, HD= hepatic dysfunction,

Table 4S: Diagnostic or endoscopic retrograde cholangiopancreatography (ERCP)-guided treatments within 3 months from MR imaging

	Method	Potential Functional Stricture on T1-MRC	Dominant stricture on T2-MRCP	High-Grade Stricture on T2-MRCP
**All ERCP**	**Sensitivity**	94%	83%	57%
	**Specificity**	90%	43%	77%
	**Positive Predictive Value**	79%	35%	48%
	**Negative Predictive Value**	98%	87%	83%
	**Accuracy**	91%	53%	71%
**ERCP-guided treatments**	**Sensitivity**	100%	81%	58%
	**Specificity**	89%	41%	76%
	**Positive Predictive Value**	74%	30%	43%
	**Negative Predictive Value**	100%	87%	85%
	**Accuracy**	91%	50%	71%
